# Effect of Two Different Tranexamic Acid Doses on Blood Loss in Head and Neck Cancer Surgery: A Randomized, Double-Blind, Controlled Study

**DOI:** 10.7759/cureus.20190

**Published:** 2021-12-05

**Authors:** Mittapalli J Babu, Praveen K Neema, Habib M Reazaul Karim, Samarjit Dey, Ripudaman Arora

**Affiliations:** 1 Anaesthesiology, Critical Care, and Pain Medicine, All India Institute of Medical Sciences, Raipur, IND; 2 Cardiac Anaesthesia, UN Mehta Institute of Cardiology and Research Centre, Ahmedabad, IND; 3 Cardiac Anaesthesiology, Sri Sathya Sai Sanjeevani Hospitals, Raipur, IND; 4 Anesthesia, Critical Care, and Pain Medicine, All India Institute of Medical Sciences Raipur, Raipur, IND; 5 Otorhinolaryngology, All India Institute of Medical Sciences, Raipur, IND

**Keywords:** pharmacotherapy, surgery, cancer, blood coagulation, hemorrhage

## Abstract

Background and aim

Head and neck cancer is frequent, and surgeries pose more significant morbidity and mortality due to multitudinal causes; heavy blood loss and transfusion are among them. Tranexamic acid (TXA) is known to stabilize the micro clots hence controlling excessive blood loss. The present study aimed to compare perioperative blood loss with two different doses of TXA and placebo to find the effectiveness and optimal dose.

Methods

With ethical approvals and informed consent, the present prospective, randomized, double-blind, controlled study was conducted in a teaching institute from May 31, 2018, to Dec 28, 2019. Patients undergoing elective head and neck cancer (HNC) surgeries were included. Preoperative Hb < 7 gm% or > 16 gm%, known coagulopathy, anticoagulant therapy, contraindications to TXA, intraoperative torrential or blood loss due to arterial injury were excluded. Group T-1 received TXA 10mg/kg, T-II received 15 mg/kg, while the control group (Gr-C) received equal volume normal saline. Data about demography, surgical time, intraoperative and postoperative blood loss, and transfusion were collected and compared. SPSS software was used for analysis; p-value <0.05 was considered significant.

Results

Ninety patients were screened, 84 completed the study. All three groups were similar in demographics. The median blood loss with 25^th ^-75^th^ percentile in group C, T-I, and T-II groups were 762.5 (513.5-1193), 541.5 (296.5-787), and 536.0 (180.5 - 879) mL, respectively; p: 0.025. There was a significant difference between the control group and T-I (p-value: 0.0153), and control and T-II (p-value: 0.0248), but an insignificant difference between T-I and T-II (p-value: 0.706). 5 (17.85%) in each of T-I and T-II required transfusion, whereas 14 (50%) in the control group required it; p < 0.011). No major clinically significant related to study drugs were noted.

Conclusion

Compared to placebo (normal saline), preoperative administration of TXA in bolus significantly reduced perioperative blood losses and transfusion requirement in patients undergoing HNC surgery as estimated using the Hb-based method. A bolus dose of doses of 10mg/kg and 15 mg/kg is equally effective.

## Introduction

Globally, head and neck cancers (HNC) are the sixth most common cancer, while in the Indian subcontinent, the prevalence is higher [1,2]. The prevalence in India is as high as 30%-40% for malignant diseases of the body [3]. Further, the etiology, clinical presentation, and patient characteristics make HNC in India unique [4]. Wide local excision and radical neck dissection have been recognized as an interwoven part of the HNC surgical procedures [3]. Bleeding is a challenge for surgeons and anesthesiologists, and often blood and blood products transfusion is required. On the other hand, the administration of blood and blood products carries multiple risks and adverse effects [5].

There are various methods employed to reduce blood loss. Antifibrinolytic agents like tranexamic acid (TXA) are one of them. It competitively blocks the lysine-binding site of plasminogen, plasmin, and tissue plasminogen activator, preventing their association with fibrin [6]. Thus, plasminogen to plasmin conversion is slowed down, and the proteolytic action of plasmin on fibrin monomers and fibrinogen is prevented. As cancer cells increase fibrinolytic activity as urokinase‑type plasminogen activator, tPA and PAI‑1 are expressed on their surface [7], the use of TXA for blood loss reduction appears logical.

However, clinical studies done in a different group of patients show variable results [8,9]. Even the blood loss assessment varies a lot. The present study aimed to evaluate the efficacy of a single preoperative bolus dose of TXA on reduction in blood loss measured from fall in hemoglobin and red blood cell transfusion in patients undergoing HNC surgery and its gross adverse effects any. Further, we compared two different doses of TXA to find a better single-dose regimen. 

## Materials and methods

The present prospective, randomized, double-blind, controlled, parallel-arm study was conducted in central India's apex level teaching hospital. The study followed the Good Clinical Practice guideline and Declaration of Helsinki; Institutional Research Cell and Ethics Committee approved the study and was prospectively registered in the clinical trial registry of India. Informed written consent was obtained from all participants. The study was conducted in the operating room and critical care unit settings from May 31, 2018, to December 28, 2019. Patients aged 18-65 of either male or female gender having a body mass index of 18.5-29.99 kg/m^2^ belonging to the American Society of Anesthesiologists physical class up to III undergoing elective Head and Neck Cancer surgeries were included. Patients having preoperative Hb < 7 gm% or > 16 gm%, known coagulopathy, or on anticoagulant therapy, or having contraindications to TXA were excluded. Further, intraoperative torrential blood loss due to arterial injury was also excluded. Preoperative coagulation profiles and serum calcium levels were also tested in all patients and optimized to an acceptable range when required. 

After identifying prospective participants and taking written and informed consent from the patients, participants were randomly allocated into groups: T-I (TXA 10 mg/kg mixed in NS to reach maximum volume 20 mL), T-II (TXA 15 mg/kg mixed in NS to reach maximum volume 20 mL) and group C (NS 20 mL). Randomization to various groups was done using a software-generated random number spreadsheet (created using http://www.openepi.com). Allocation concealment was done by keeping the random sheet centralized and was not disclosed to either participant or investigators. The person preparing the study drug and coded was not involved in data collection, thus the patient and data collector including the surgeon and anesthesiologists were kept blind to the intervention. 

The patients were premedicated with 1 mg midazolam and 0.2 mg glycopyrrolate in the preoperative holding area. The study drug/placebo was prepared as per random allocation by a resident doctor who is not a part of the study. The allocated drug was administered intravenously over 20 minutes by the resident who prepared it in the preoperative holding area and marked them as a study group with codes. After that, patients were shifted to the operating room. General anesthesia was induced with 1.5-2 mg/kg propofol and fentanyl two mcg/kg. Endotracheal intubation was facilitated by vecuronium 0.1 mg/kg. Anesthesia was maintained with oxygen, nitrous oxide (titrated to deliver at least 40% inspired O2), and isoflurane to achieve 1.1 + 0.1 MACage [10]. Patients were ventilated to EtCO2 of 34 ± 3 mmHg. The patients' MAP (mean arterial pressure) was maintained within ±20% of baseline but not below 60 mmHg for more than 10 minutes at a stretch; vasoactive drugs were used as per clinical judgment. Preoperative, pretransfusion, and postoperative hemoglobin (Hb) were measured, and blood loss was calculated using Hb based method. The following formula was used for calculating the actual blood loss (ABL); ABL = [estimated blood volume (EBV) (Hbi-Hbf / Hbav)], where Hbi: initial Hb; Hbf: final or pretransfusion Hb; Hbav: average of initial and final Hb [11]. Blood samples were analyzed using a colorimetric method for Hb estimation. The drain amount was noted at 24 hours and 48 hours post-operatively. Other parameters collected as data were clinic-demographic details, duration of surgery, Intra-operative IV fluids infused, heart rate, mean arterial pressure.

The present study was based on the previous meta-analysis findings where it was found that TXA reduces blood loss and blood transfusions by 38%. The sample size is calculated for 80% power (1-β) with absolute precision of 5% (1-α = 95%). Online epidemiological tool Open Epi (Open-Source Epidemiologic Statistics for Public Health; http://www.openepi.com/SampleSize/SSCohort.htm) was used for calculating the sample size (Fleiss method with continuity correction); a sample size of 21 patients in each group was required. Considering the study's randomized nature, the design effect of 1 was applied, and 15% attrition was added to reach a final sample size of at least 25 patients per group.

Data collection was done in a pre-approved case record form, and a master chart was prepared using Microsoft Excel. Analysis of variance, Kruskal Wallis, Mann-Whitney, and chi-square tests was used as applicable to compare the groups, and p-value <0.05 was considered significant. The results are analyzed by IBM SPSS version 24.0 Software.

## Results

**Figure 1:**
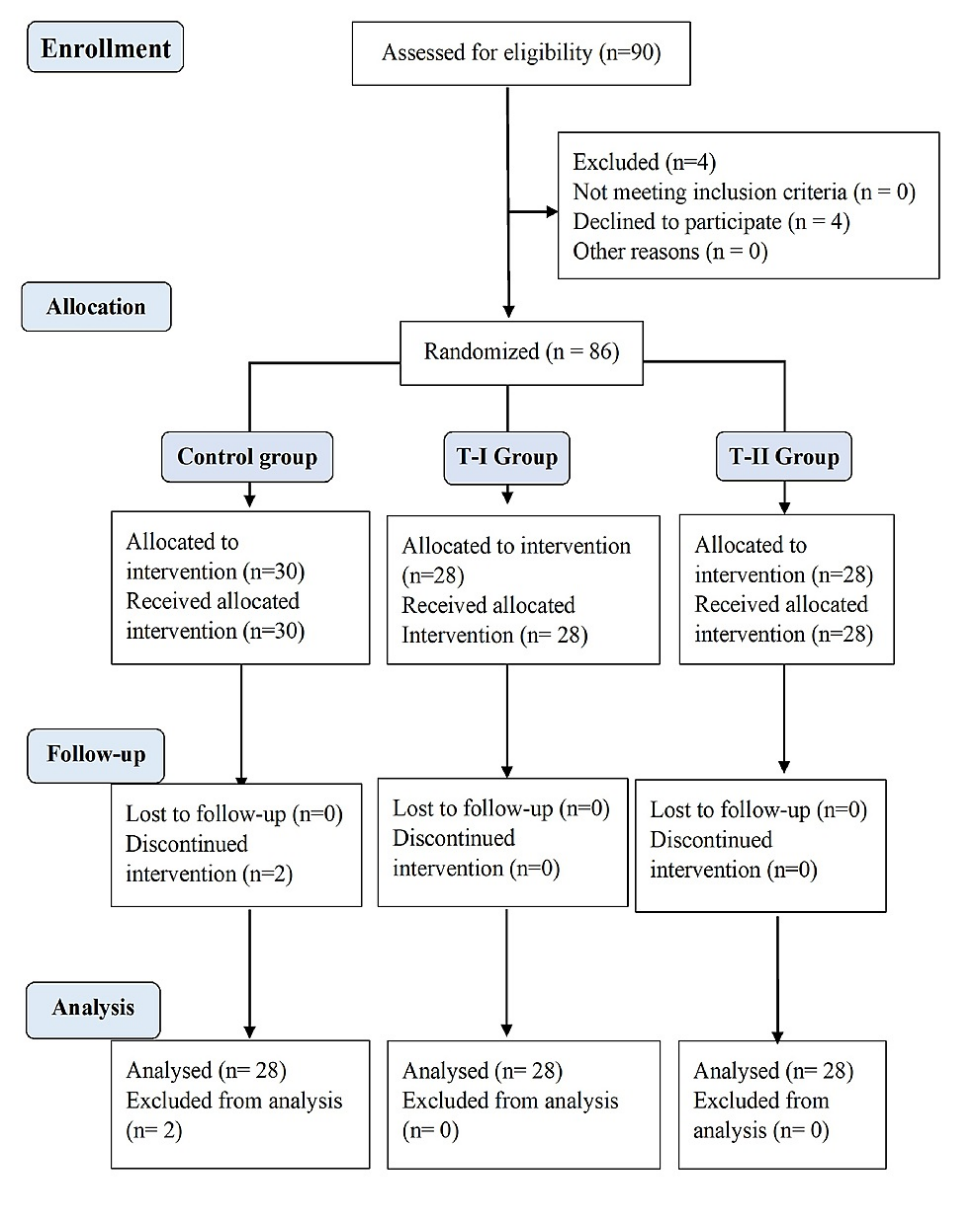
CONSORT flow diagram. CONSORT: Consolidated Standards of Reporting Trials.

Ninety patients were screened for eligibility, and following exclusions, data from 84 participants were analyzed (Figure 1).

The demographic parameters, preoperative hemodynamics, preoperative hemoglobin, and maximum allowable blood loss were similar and presented in Table 1. None of the patients were severely hypocalcemic and entire patients underwent wide local excision of the lesions with radical neck dissection and free flap reconstruction for carcinoma oral cavity; differences were statistically insignificant. 

**Table 1:** Clinicodemographic data expressed as mean + standard deviation or absolute number and their comparison tested using analysis of variance. BMI: body mass index; ASA-PS: American Society of Anesthesiologists physical status class; Hb: hemoglobin; MABL: maximum allowable blood loss; MAP: mean arterial pressure; HR: heart rate. *Data in absolute number.

Parameters	Control (N=28)	T-I (N=28)	T-II (N=28)	p-value
Age (years)	44.29 ± 11.74	47.21 ± 9.30	49.00 ± 9.08	0.218
Weight (kilogram)	58.85 + 13.23	59.51 + 10.27	56.12 + 11.28	0.517
Height (centimetre)	160.04 ± 8.46	162.50 ± 7.87	159.89 ± 7.69	0.396
BMI (kg/m^2^)	22.8 + 3.73	22.58 + 3.79	21.98 + 3.48	0.687
Gender (Female:Male)*	8: 20	4: 24	7: 21	0.413
ASA-PS (II: III)*	25: 3	25: 3	26: 2	0.871
Pre-op Hb (gm/dl)	12.91 + 1.79	13.72 + 1.4	13.61 + 1.76	0.146
MABL (mL)	1491.7 ± 446.3	1704.1 ± 408.7	1600.7 ± 555.9	0.252
Pre-op MAP (mmHg)	95.54 ± 13.39	98.64 ± 14.81	94.75 ± 11.87	0.520
Pre-op HR (beats/min)	88.79 ± 14.06	86.57 ± 15.17	83.50 ± 16.53	0.434

The mean arterial pressure during the surgery was also similar except for immediately after induction when the treatment groups were having lower blood pressures (Table 2).

**Table 2:** Comparison of mean arterial pressure of the three groups tested using one-way analysis of variance. SD: standard deviation, n: number of participants at that particular time points.

Time points	Control Mean ± SD	T-I Mean ± SD	T-II Mean ± SD	p-value
Baseline	95.54 ± 13.39	98.64 ± 14.81	94.75 ± 11.87	0.520
20 Mins	112.25 ± 14.75	92.07 ± 14.40	87.96 ± 13.62	0.000
40 Mins	86.75 ± 10.49	89.50 ± 12.46	84.82 ± 15.82	0.410
60 Mins	86.57 ± 12.86	83.04 ± 11.06	81.64 ± 12.90	0.308
80 Mins	83.29 ± 12.10	84.82 ± 14.78	83.71 ± 13.63	0.909
100 Mins	81.71 ± 11.73	84.07 ± 10.99	82.54 ± 11.00	0.726
120 Mins	81.50 ± 10.45	81.35 ± 09.07	81.79 ± 10.44	0.987
140 Mins	81.86 ± 10.57	81.11 ± 09.06	82.75 ± 11.69	0.842
160 Mins	84.96 ± 15.54	82.21 ± 10.29	81.21 ± 10.98	0.511
180 Mins	81.50 ± 14.42	92.79 ± 63.74	81.93 ± 12.28	0.463
200 Mins	80.89 ± 11.64	83.50 ± 08.48	78.71 ± 08.97	0.194
220 Mins	81.93 ± 10.58	81.79 ± 08.84	78.68 ± 09.66	0.373
240 Mins	80.46 ± 11.06	80.64 ± 10.81	78.61 ± 07.51	0.698
260 Mins (n =80)	82.39 ± 08.99	78.86 ± 09.25	79.13 ± 07.89	0.257
280 Mins (n =80)	80.64 ± 06.72	80.46 ± 09.80	78.29 ± 07.80	0.531
300 Mins (n = 78)	80.54 ± 08.87	80.38 ± 10.52	77.25 ± 07.00	0.348
320 Mins (n = 75)	81.14 ± 08.20	80.42 ± 10.80	76.05 ± 12.55	0.211
340 Mins (n = 72)	80.61 ± 08.15	77.96 ± 09.26	77.74 ± 07.70	0.408
360 Mins (n = 70)	81.58 ± 10.21	79.92 ± 08.59	76.21 ± 06.72	0.133
380 Mins (n = 67)	81.79 ± 10.01	77.42 ± 08.05	79.79 ± 12.06	0.323
400 Mins (n = 63)	82.74 ± 12.04	75.81 ± 06.53	80.84 ± 09.78	0.064
420 Mins (n = 56)	80.83 ± 11.24	75.88 ± 06.04	79.12 ± 09.19	0.276
440 Mins (n = 54)	80.62 ± 10.41	79.41 ± 09.79	79.19 ± 10.08	0.896
460 Mins (n = 50)	80.95 ± 09.95	78.73 ± 11.57	78.00 ± 09.97	0.682
480 Mins (n = 47)	81.85 ± 11.48	77.38 ± 10.57	77.71 ± 11.43	0.437
500 Mins (n = 39)	80.94 ± 10.71	72.67 ± 05.81	75.33 ± 06.89	0.055
520 Mins (n = 37)	77.25 ± 11.26	77.00 ± 10.33	75.42 ± 07.55	0.882
540 Mins (n = 34)	79.07 ± 09.79	77.75 ± 08.83	75.91 ± 07.16	0.668
560 Mins (n = 32)	79.29 ± 08.70	79.25 ± 07.85	76.10 ± 06.81	0.584
580 Mins (n = 26)	76.09 ± 09.17	80.43 ± 08.98	77.13 ± 06.62	0.566
600 Mins (n = 23)	80.22 ± 09.42	81.43 ± 07.70	75.43 ± 05.74	0.341
620 Mins (n = 20)	77.88 ± 07.57	81.60 ± 09.21	79.00 ± 04.66	0.663
640 Mins (n = 18)	79.00 ± 10.37	85.67 ± 10.07	77.86 ± 08.47	0.499
660 Mins (n = 18)	78.88 ± 11.10	81.67 ± 08.32	77.29 ± 11.30	0.843
680 Mins (n =14)	74.33 ± 08.76	81.50 ± 07.78	77.00 ± 11.33	0.677
700 Mins (n = 14)	81.00 ± 10.83	85.00 ± 14.14	76.00 ± 08.30	0.509
720 Mins (n = 13)	85.60 ± 14.42	85.50 ± 14.85	76.00 ± 08.10	0.378
740 Mins (n = 12)	89.00 ± 19.41	84.00 ± 14.14	76.67 ± 09.07	0.416
760 Mins (n =11)	87.25 ± 16.86	91.00 ± 02.82	77.80 ± 07.29	0.341
780 Mins (n = 10)	77.00 ± 06.56	82.50 ± 04.95	74.80 ± 06.72	0.409
800 Mins (n = 8)	79.00 ± 07.21	79.00 ± 0.00	70.50 ± 04.80	0.219
820 Mins (n = 8)	79.00 ± 08.72	85.00 ± 0.00	72.50 ± 05.26	0.282
840 Mins (n = 7)	82.50 ± 13.44	81.00 ± 0.00	75.25 ± 03.78	0.543
860 Mins (n = 7)	77.50 ± 12.02	80.00 ± 0.00	74.50 ± 02.52	0.719
880 Mins (n = 7)	71.50 ± 03.54	81.00 ± 0.00	75.00 ± 02.31	0.103
900 Mins (n = 7)	78.50 ± 12.02	77.00 ± 0.00	57.50 ± 38.46	0.746
920 Mins (n = 6)	83.00 ± 0.00	78.00 ± 0.00	58.00 ± 39.10	0.811
940 Mins (n = 3)	92.00 ± 0.00	79.00 ± 0.00	73.00 ± 0.00	0.000
960 Mins (n = 2)	————	82.00 ± 0.00	72.00 ± 0.00	———-

An almost similar trend was also noted for heart rate among the groups (Table 3) throughout the surgery.

**Table 3:** Comparison of heart rates of the three groups tested using one-way analysis of variance. SD: standard deviation; n: number of participants at that particular time points.

Time points	Control Mean ± SD	T-I Mean ± SD	T-II Mean ± SD	p-value
Baseline	88.79 ± 14.06	86.57 ± 15.17	83.50 ± 16.53	0.434
20 Mins	95.79 ± 12.55	89.50 ± 12.31	86.36 ± 12.98	0.021
40 Mins	91.21 ± 14.36	91.21 ± 14.42	88.93 ± 13.38	0.782
60 Mins	88.93 ± 12.01	87.93 ± 11.44	86.50 ± 12.76	0.752
80 Mins	84.00 ± 10.93	90.14 ± 15.34	83.36 ± 13.85	0.122
100 Mins	80.64 ± 18.15	86.21 ± 13.14	78.50 ± 09.58	0.113
120 Mins	81.29 ± 11.88	83.64 ± 13.47	79.43 ± 09.33	0.405
140 Mins	78.43 ± 10.33	81.58 ± 11.08	78.43 ± 09.61	0.427
160 Mins	77.50 ± 11.76	80.36 ± 10.89	75.86 ± 08.98	0.281
180 Mins	77.86 ± 11.17	78.86 ± 08.04	74.64 ± 08.56	0.218
200 Mins	77.00 ± 11.64	75.29 ± 08.91	75.50 ± 08.14	0.771
220 Mins	77.00 ± 11.58	75.86 ± 10.37	77.21 ± 08.40	0.867
240 Mins	74.64 ± 11.00	76.00 ± 09.12	76.14 ± 10.14	0.829
260 Mins (n =80)	76.50 ± 10.52	75.43 ± 10.02	75.92 ± 06.70	0.911
280 Mins (n =80)	75.79 ± 09.92	73.33 ± 09.15	74.25 ± 08.41	0.610
300 Mins (n = 78)	75.43 ± 09.36	72.54 ± 08.92	72.82 ± 07.70	0.417
320 Mins (n = 75)	77.36 ± 09.30	74.56 ± 07.95	73.20 ± 09.05	0.247
340 Mins (n = 72)	74.65 ± 08.61	72.56 ± 07.97	73.70 ± 07.85	0.652
360 Mins (n = 70)	74.60 ± 08.22	73.39 ± 07.07	73.05 ± 08.85	0.792
380 Mins (n = 67)	76.70 ± 09.26	74.09 ± 07.32	73.06 ± 07.72	0.346
400 Mins (n = 63)	76.64 ± 07.59	75.27 ± 06.80	71.18 ± 06.48	0.055
420 Mins (n = 56)	77.64 ± 09.65	75.26 ± 08.22	70.35 ± 06.94	0.034
440 Mins (n = 54)	77.14 ± 11.07	76.35 ± 07.69	69.38 ± 09.26	0.041
460 Mins (n = 50)	77.47 ± 11.25	79.57 ± 10.23	68.40 ± 08.01	0.009
480 Mins (n = 47)	78.00 ± 10.43	79.80 ± 07.39	71.00 ± 09.01	0.066
500 Mins (n = 39)	77.88 ± 09.70	77.78 ± 06.96	72.33 ± 08.61	0.217
520 Mins (n = 37)	78.27 ± 09.82	77.56 ± 09.04	75.17 ± 09.55	0.695
540 Mins (n = 34)	80.57 ± 12.05	75.33 ± 06.25	72.55 ± 08.95	0.133
560 Mins (n = 32)	76.00 ± 11.47	72.00 ± 06.76	70.86 ± 06.20	0.457
580 Mins (n = 26)	75.78 ± 15.11	70.57 ± 08.77	74.00 ± 07.90	0.680
600 Mins (n = 23)	77.11 ± 13.72	76.67 ± 14.24	77.33 ± 06.65	0.995
620 Mins (n = 20)	81.25 ± 18.97	71.00 ± 07.39	80.33 ± 06.38	0.471
640 Mins (n = 18)	86.00 ± 22.60	67.33 ± 06.11	81.67 ± 03.88	0.261
660 Mins (n = 18)	79.67 ± 19.53	63.00 ± 04.24	77.67 ± 06.12	0.359
680 Mins (n =14)	70.80 ± 05.40	66.00 ± 02.83	75.67 ± 08.80	0.259
700 Mins (n = 14)	73.60 ± 7.40	77.00 ± 07.07	72.40 ± 05.55	0.716
720 Mins (n = 13)	75.60 ± 08.65	79.00 ± 01.41	72.40 ± 06.23	0.539
740 Mins (n = 12)	71.00 ± 01.41	79.00 ± 04.24	69.00 ± 04.76	0.097
760 Mins (n =11)	70.00 ± 02.83	82.00 ± 05.65	68.67 ± 02.31	0.031
780 Mins (n = 10)	68.00 ± 02.83	72.00 ± 0.00	68.00 ± 06.93	0.834
800 Mins (n = 8)	66.00 ± 02.83	78.00 ± 0.00	77.33 ± 11.37	0.467
820 Mins (n = 8)	65.00 ± 04.24	78.00 ± 0.00	72.67 ± 15.53	0.708
840 Mins (n = 7)	67.00 ± 09.90	78.00 ± 0.00	60.00 ± 05.66	0.374
860 Mins (n = 7)	80.00 ± 11.31	76.00 ± 0.00	58.00 ± 05.66	0.234
880 Mins (n = 7)	76.00 ± 08.49	76.00 ± 0.00	68.00 ± 0.00	0.484
900 Mins (n = 7)	79.00 ± 01.41	80.00 ± 0.00	66.00 ± 0.00	0.009
920 Mins (n = 6)	75.00 ± 04.24	——	60.00 ± 0.00	0.212
940 Mins (n = 3)	78.00 ± 0.00	——	62.00 ± 0.00	——
960 Mins (n = 3)	70.00 ± 02.83	——	66.00 ± 0.00	0.454

**Figure 2:**
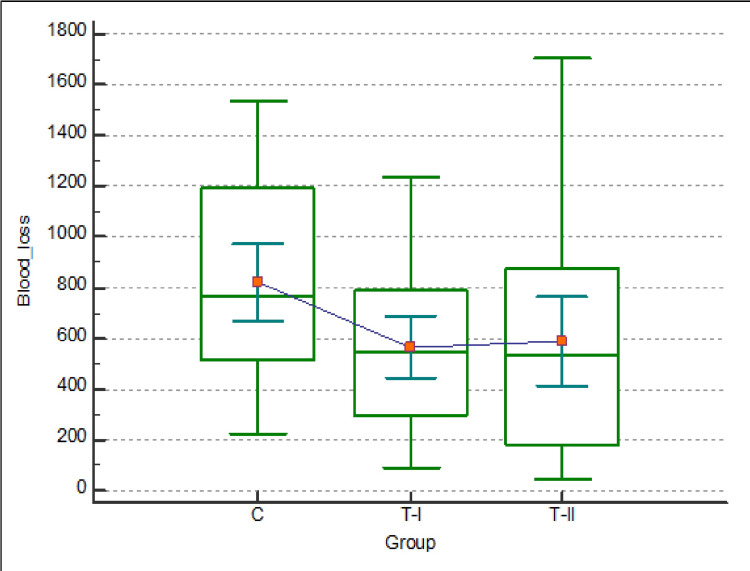
Showing the median, 75th and 25th percentile blood loss among the groups.

Intraoperative median blood loss in group C, T-I, and T-II groups was 762.5 mL, 541.5 mL, and 536.0 mL, respectively, with a p-value of 0.025 (Figure 2).

The TXA groups had significantly lower blood loss when compared to the control (normal saline) group, but there was an insignificant difference between T-I & T-II (p-value: 0.706). Two patients in the control group required rapid blood transfusion due to intraoperative sudden blood loss of around 500 mL over five minutes; hence, they were excluded. Among the rest, the need for blood transfusion was still higher in the control group (50% versus 17.86%) than in either of the TXA group. There was no difference in the transfusion requirement among the two TXA groups (17.86% versus 17.86%; p: 1.00). The mean surgical durations were also similar. Even the drain amount was significantly higher in the control group at 24h and 48h (Table 4). No clinically significant adverse events were noted.

**Table 4:** Comparison of surgical duration, blood loss, drain and fluid therapy tested using Kruskal Wallis Test, #Mann Whitney test, and @Chi-square test. IQR: interquartile range, $data in mean and standard deviation.

Parameters	Control Median (IQR)	T-I Median (IQR)	T-II Median (IQR)	p-value
Duration of surgery (min)	462.50 (199.0)	360.00 (201.3)	379.50 (239.75)	0.125
Intraoperative Blood Loss (mL)	762.5 (679.5)	541.5 (490.5)	536.0(698.5)	C vs T-1 0.01^#^ C vs T-II 0.02^#^ T-I vs T-II 0.70^#^
24 hours drain volume (mL)	60.00 (83.0)	41.00 (26.5)	30.00 (23.00)	0.006
48 hours drain volume (mL)	79.00 (92.8)	40.00 (30.0)	34.00 (43.75)	0.001
Blood transfusion (number)	14 (50%)	5 (17.86%)	5 (17.86%)	0.011^@^
Intravenous fluid (mL)^$^	2411.4 + 876.4	1932.1 + 1032.5	1821.1 + 1063.7	0.067

## Discussion

The present study results indicate that the use of TXA reduces perioperative blood loss significantly, thereby reducing the need for blood transfusion compared to placebo (NS). However, the difference between the two tranexamic acid-treated groups (10 and 15 mg/kg) regarding intraoperative and postoperative blood loss and blood transfusions administered were indifferent. The reduction in blood loss in our cohort is attributable to the use of TXA because patient characteristics and surgical duration were similar. Preoperative Hb levels and their maximum allowable blood loss were also statistically indifferent in all three groups. Additionally, the patients are operated on in a short span of one and half-year; during this period, surgical techniques have remained unchanged, and the patients were operated on by the same group of surgeons, including the lead surgeon. The blood transfusion practice has also remained similar during this period.

TXA has been used by many researchers to evaluate the impact on intraoperative blood loss and postoperative blood transfusion requirement in patients undergoing major surgeries like cardiac, orthopedics, and head and neck cancer surgery and found variable results [8,9,12]. One of the frequently observed non-homogeneity among the studies is the methods of blood loss estimation. There are different methods of blood loss estimation practiced the world over [13]. Although the methods correlate relatively well, it is not devoid of concern, as under or overestimation can lead to mismanagement of patient in the perioperative period, the clinician might end up in unnecessary blood transfusion or crystalloids infusion. The Hb-based method gives an objective way of finding the blood loss compared to an analog visual method, and the risk of handling blood is also avoided. Further, visual estimations tend to estimate the blood loss lower as compared to formula estimation. [13] In a mixed-effect meta-analysis, the colorimetric method of blood loss estimation has been reported to have the highest correlation and least bias [14]. Nevertheless, the patients in the control group received slightly higher volume (p: 0.067) of intravenous fluids (difference of mean volume approximately 500 mL over 8h of average durations). It is partly explained by the approximately 1-1.5h prolonged surgical duration (p: 0.125) and also was probably to maintain intravascular volume and hemodynamics in the face of increased intraoperative blood loss. However, it is noteworthy that an overzealous fluid administration to maintain intravascular volume results in haemodilution, decreasing the Hb measured and triggering blood transfusion. Therefore, it is possible that a few blood transfusions could have been avoided in the control group. The difference found in our study compared to the recent studies and even a relatively older meta-analysis is probably explained by the above facts [8,15].

On the other hand, blood transfusion requirement was similar in the two groups receiving tranexamic acid 10 mg/kg and 15 mg/kg, suggesting 10 mg/kg to be an appropriate dose of tranexamic acid for reducing blood loss. This dose-response relationship correlates to a previously reported study where the authors used TXA in patients undergoing cardiac operation with extracorporeal circulation [16]. Literature indicates the use of 10-30 mg/kg TXA as a bolus, and even 5-10 mg/kg/h as maintenance. Although these dosages are well tolerated clinically, our findings indicate that 10 mg/kg bolus is sufficient. 

Further, TXA has been reported to induce microvascular coagulation, but the risk is low in bleeding patients [17]. However, it has a potential effect on causing seizures in a dose-dependent manner [17]. In such a background, and as per commonly prevailing good practice norms, a minimum effective dose of the drug should be used. Although we have observed clinically attributable adverse effects of TXA, our study was limited because we have not evaluated the effect through laboratory-based diagnostic aids for microvascular coagulation effects.

The other limitation of our study is that it is a single-center study; the sample size, although it was well-powered to detect a significant difference in the blood loss, the post-hoc analysis for transfusion received showed a power of 71%. The surgical duration and the effect time evaluated, i.e., up to 48h, were higher, and a single bolus dose of TXA is unlikely to provide a therapeutic effect for such a period. Therefore, bolus followed by infusion or intermittent dose might be a good option which we have not explored.

Even so, given that the highly significant difference in the blood loss and transfusion observed was highly significant, the possibility of nullifying the effect of TXA on reducing the blood loss and transfusion by the biases mentioned earlier is remote. Furthermore, considering the vast range of infectious and even non-infectious complications of blood transfusion, including increased rates of recurrence in various malignancies, volume overload, lung injuries [5,18], and in the absence of clinically significant adverse effects of TX; administration of 10mg/kg as a bolus before incision can be considered as good clinical practice.

## Conclusions

Compared to placebo (normal saline), preoperative administration of TXA in bolus significantly reduced perioperative blood losses and transfusion requirement in patients undergoing HNC surgery as estimated using the Hb-based method. A bolus dose of 10 mg/kg and 15 mg/kg is equally effective in reducing the blood loss and need for blood transfusion. However, we will require further study to confirm the findings especially the in context of the number of blood transfusions.
